# Patients Using an Online Forum for Reporting Progress When Engaging With a Six-Week Exercise Program for Knee Conditioning: Feasibility Study

**DOI:** 10.2196/rehab.8567

**Published:** 2018-04-26

**Authors:** Philip Bright, Karen Hambly

**Affiliations:** ^1^ School of Sport and Exercise Sciences Medway Campus University of Kent Chatham United Kingdom; ^2^ Research Department European School of Osteopathy Maidstone United Kingdom; ^3^ School of Sport and Exercise Sciences Medway Campus University of Kent Maidstone United Kingdom

**Keywords:** eHealth; social media; exercise therapy; rehabilitation

## Abstract

**Background:**

The use of electronic health (eHealth) and Web-based resources for patients with knee pain is expanding. Padlet is an online noticeboard that can facilitate patient interaction by posting virtual “sticky notes.”

**Objective:**

The primary aim of this study was to determine feasibility of patients in a 6-week knee exercise program using Padlet as an online forum for self-reporting on outcome progression.

**Methods:**

Undergraduate manual therapy students were recruited as part of a 6-week study into knee conditioning. Participants were encouraged to post maximum effort readings from quadriceps and gluteal home exercises captured from standard bathroom scales on a bespoke Padlet. Experience and progression reporting were encouraged. Posted data were analyzed for association between engagement, entry frequency, and participant characteristics. Individual data facilitated single-subject, multiple-baseline analysis using statistical process control. Experiential narrative was analyzed thematically.

**Results:**

Nineteen participants were recruited (47%, 9/19 female); ages ranged from 19 to 53 years. Twelve individuals (63%) opted to engage with the forum (range 4-40 entries), with five (42%) reporting across all 6 weeks. Gender did not influence reporting (odds ratio [OR] 0.76, 95% CI 0.06-6.93). No significant difference manifested between body mass index and engagement *P*=.46); age and entry frequency did not correlate (R^2^=.054, 95% CI –0.42 to 0.51, *P*=.83). Statistically significant conditioning profiles arose in single participants. Themes of pain, mitigation, and response were inducted from the experiences posted.

**Conclusions:**

Patients will engage with an online forum for reporting progress when undertaking exercise programs. In contrast to related literature, no significant association was found with reporting and gender, age, or body mass index. Individual posted data allowed multiple-baseline analysis and experiential induction from participants. Conditioning responses were evident on visual inspection. The importance of individualized visual data to patients and the role of forums in monitoring patients’ progress in symptomatic knee pain populations need further consideration.

## Introduction

The use of Web-based resources and eHealth apps for patients with knee pain is an area of expansion [1,2]. The term eHealth encompasses technology delivered through computer, hand-held tablet, or mobile phone that supports patients and practitioners in decision making, coping strategies, treatment approaches, or functional improvement [3]. There are a range of knee conditions such as osteoarthritis, arthroplasty, and cruciate ligament tears that are being informed by patient decision aids, electronic patient-reported outcomes, and biofeedback software [4-6]. Positive effects are noted across a range of conditions including knee osteoarthritis, but further work is required on determining suitable interactions between patients and these eHealth measures [7,8].

The cost of developing and delivering eHealth resources is considered to be offset by the ease of patient accessibility [9]. The lack of quality studies and the heterogeneous nature of conditions supported by eHealth prevent full unequivocal endorsement of the cost-effectiveness of technology-driven approaches [10,11]. The expedient delivery and low-cost development afforded by Web 2.0 apps may facilitate further access to eHealth [12] and wider health information technology [13], including patient-reported health records [14]. The Web 2.0 platform has increased participation through social media and the sharing of experience due to the ease of posting materials such as video files and online forums [15]. This latest generation of Internet development is seen as providing a collaborative medium for knowledge generation and dissemination [16]. This aligns to the potential interactive nature of eHealth programs that has been reported to facilitate health care engagement [17].

Educational research and pedagogic practice have been fruitful areas of exploration around Web 2.0 apps [18]. The option to motivate learners in ever more expansive ways of engagement adds to the wider participation aspirations of higher education [19]. There are a range of tools that allow for students to engage in learning and feedback in the Web 2.0 toolset that may have applicability in eHealth [18,20,21]. These tools have been deployed to support chronic conditions in older adults with regards to education and self-management; the pedagogue/student relationship transformed to clinician/patient with the shared aim of empowerment [22]. The exposure to the range of eHealth has been seen to bridge gender and age differences, but there is a suggestion that gender influences engagement with Web 2.0 apps [23]. Online social interaction has also been explored with respect to weight management facilitated through discussion boards; attrition rates are reportedly high in this area and little change is noted in body mass index (BMI) as a common outcome measure [24]. High BMI has been seen to be associated with higher attrition rates.

Padlet is a Web 2.0 online noticeboard that can be used to facilitate participant interaction by posting of multimedia files as virtual “sticky notes” with mediation by an administrator [25]. The scope for using this resource as an eHealth app has been investigated with some success in terms of engaging surgeons or clinicians to discuss cases in a forum setting [12]. The initial disadvantages described around mobile access have been addressed with the latest software release [26]. There is potential that this platform could facilitate an online health community; online health communities can be used to share patient and clinical experiences while disseminating expert-moderated knowledge [27]. These communities have the potential to allow patients to report progress and responses that are normally qualitative in nature [28]. With the range of biofeedback devices now available, the sharing of quantitative data to monitor patient progress and motivation via Web 2.0 apps has potential to influence compliance [29]. The use of the Padlet Web 2.0 platform to facilitate a patient-led, clinician-moderated online forum around knee conditioning exercises with biofeedback data has not been explored. The potential to use this type of forum for participant-specific primary data gathering is also an area requiring further investigation.

The aim of this study was to determine the feasibility of patients engaging with an online forum to report progress using biofeedback as part of a 6-week exercise program to improve knee function.

The primary objective was to facilitate a moderated, online community and explore participant characteristics that reportedly influence engagement, with a view to answer the following research question: is there a difference in reporting progress in an online forum based on gender, age, and BMI. A secondary objective was to ascertain if sufficient individual data were reported to complete a multiple-baseline case study for participants in the study. A tertiary objective was to establish if sufficient qualitative data were posted to allow induction of descriptive themes.

## Methods

### Design

This was a mixed-methods, quasi-experimental feasibility study with an integrated single-case, multiple-baseline, ABCD analysis and descriptive thematic summary.

### Participants

As part of a parallel study into the effects of biofeedback on knee function, participants were recruited from current year 1 to 4 undergraduate students in the osteopathy program at the European School of Osteopathy and year 2 undergraduates in the sports therapy program at the University of Kent. Recruitment took place from August 2016 to January 2017, and student participants were invited to take part in the study via email and notices placed around campus. The inclusion criteria were male and female adult students who had daily access to bathroom scales, permitted receipt of reminders via text message, and had online access via any suitable device. The exclusion criteria were if they were suffering with bilateral knee or hip pain, undertook recurrent high-intensity physical training, or had an underlying metabolic disorder or neuromuscular condition.

### Online Forum Development

**Figure 1:**
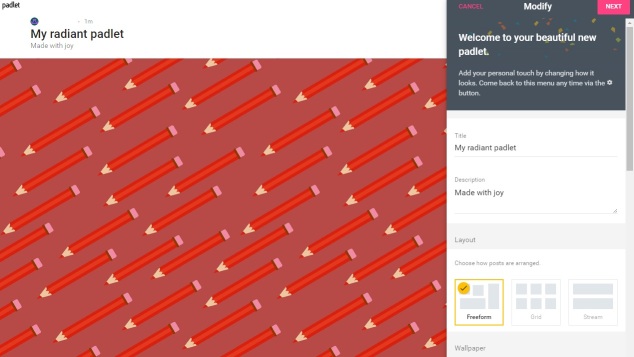
Creation page for Padlet wallpaper is indicative of background themes that can be customized.

The Padlet Web 2.0 app (Padlet Co, Sunnyvale, CA, USA) was used to develop the forum for posting of participant data; the Padlet platform facilitates multiple users sharing information and resources in a discrete environment. From the main site page, accessed via a personalized user and password, the “+make a Padlet” option was selected and a freeform option for the forum was selected as demonstrated in Figure 1.

As users were encouraged to share information and experiences. The posts were not anonymized, but oversight of the activity was conducted by the lead researchers on the study (PB, KH). A code of conduct was posted on the webpage to ensure acceptable standards of behavior were adopted. The details of this can be viewed in Textbox 1. Padlet also operates its own policy for reporting and removing inappropriate content in addition to user-defined practice available on their website.

### Procedure

**Figure 2:**
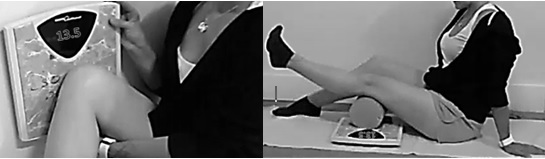
Seated clamshell and short-arc quadriceps exercise.

The following characteristic data were collected at baseline: height (cm), weight (kg), waist circumference (cm), BMI (kg/m^2^), activity levels (11-point numerical rating scale), age, and gender. Participants were inducted into a knee program consisting of staged repetitions of a seated clamshell exercise (an adaptation from Distefano et al [30]) and a short arc quadriceps extension (Figure 2). The clamshell required participants to abduct the hip, contracting gluteals as hard as possible against the resistance of bathroom scales supported against a wall. The short arc quadriceps exercise required the participant to begin with a flexed knee over a foam roller (or equivalent bolster support) resting on bathroom scales positioned on a stable surface. The exercise was completed by contracting the quadriceps to extend the leg through the shortened range, registering contraction force on the scales beneath the roller. Both required a 5-second contraction and 2-second relaxation phase.

Both exercises were repeated in sets of 12 and on both legs with a 60-second relaxation phase between sets. The progression phases are depicted in Table 1.

Participants were sent text reminders on the days they were required to perform the exercises. The text messages included a hyperlink to the bespoke Padlet forum with instructions detailing their exercise and video guidance materials (see Multimedia Appendix 1). Participants were also requested to post readings of their maximum effort in kilograms, obtained from the bathroom scales, onto the online forum after each exercise session.

**Code of conduct displayed on Padlet.**

The use of this moderated forum is to: provide information to study participants; allow a medium for recording progress; facilitate sharing of experiences during the course of the study. The exchanges should remain respectful and courteous at all times. Banter is encouraged but the study moderators policing activity will ensure any offensive or inappropriate comments or images are removed.

Participants that persist in posting such material will be asked to withdraw from the study.

**Table 1:** Exercise progression details for participants.

Week	Exercise progression	Phase
Weeks 1 and 2	Maintain 2 sets of 12 repetitions every other day	Phase A
Weeks 3 and 4	Maintain 3 sets of 12 repetitions every other day	Phase B
Week 5	Maintain 4 sets of 12 repetitions every other day	Phase C
Week 6	Maintain 5 sets of 12 repetitions every other day	Phase D

### Outcome Measures

The primary outcome measure was the number of recorded entries detailing progression with the exercise schedule. A secondary outcome measure was the maximum voluntary contraction reading as captured from the bathroom scales from each exercise session. This was provided by the participants over all stages of engagement within the study.

### Ethics

The study protocol was submitted to and approved by the Research Ethics Committees of the European School of Osteopathy and the School of Sport and Exercise Sciences, University of Kent, as part of a larger study exploring the use of biofeedback in a knee conditioning program.

### Statistical Analysis

The Padlet postings were exported to a spreadsheet and aligned to participant baseline data. Summary and inferential statistics were calculated using Excel version 16 (Microsoft Corporation, Redmond, WA, USA) and Analyse-it version 4.65.3 (Analyse-it Software, Ltd, Leeds, UK). The numbers of recorded entries and BMI were assessed for distribution and equality of variance; gender group relationships and differences in reporting were explored using odds ratios with 95% confidence intervals and the Mann-Whitney *U* test. Physical characteristics (BMI) and reporting differences were also explored using the Student *t* test. Correlation between age and recording of entries was explored using the Spearman test; statistical significance was set at *P*<.05. Entries entered against one date were considered a single entry so multiple data added under a single date were only counted once. Discrete nominal values were derived from this in terms of binary (yes/no) indication of engagement with the forum to allow proportional analysis of association.

The staged recordings of maximum voluntary isometric contractions were extracted from the forum-recorded entries and three consistent datasets were analyzed using a multiple-baseline [31], ABCD case study [32] approach aligned to four (one baseline and three progressive) stages of exercise. A statistical process control (SPC) visual analysis [33] was applied to the resultant line graphs with means and standard deviations calculated from phase A baseline data. Statistical significance was regarded as two consecutive data points outside ±2 standard deviations in phases B, C, or D. Linear trend lines were added to indicate direction of individual progress. Finally, open-forum comments were analyzed within a descriptive thematic framework [34] and summarized in relation to the source participants.

## Results

### Baseline Characteristics

A total of 19 participants were recruited. The group was 47% female (9/19); age ranged from 19 to 53 years (mean 32.79, SD 10.78 years) and BMI ranged between 16.63 and 33.83 kg/m^2^ (mean 25.02, SD 4.39 kg/m^2^); eight individuals (42%) were over the desired 25 kg/m^2^. Mean height was 173.47 (SD 10.06) cm, mean weight was 75.65 (SD 16.20) kg, and median waist circumference was 84.0 (IQR 12.7) cm. Participant’s mean activity rating was 4.42 (SD 1.30) and the median number of Padlet entries was 8 (IQR 16).

### Primary Outcome Measure

**Figure 3:**
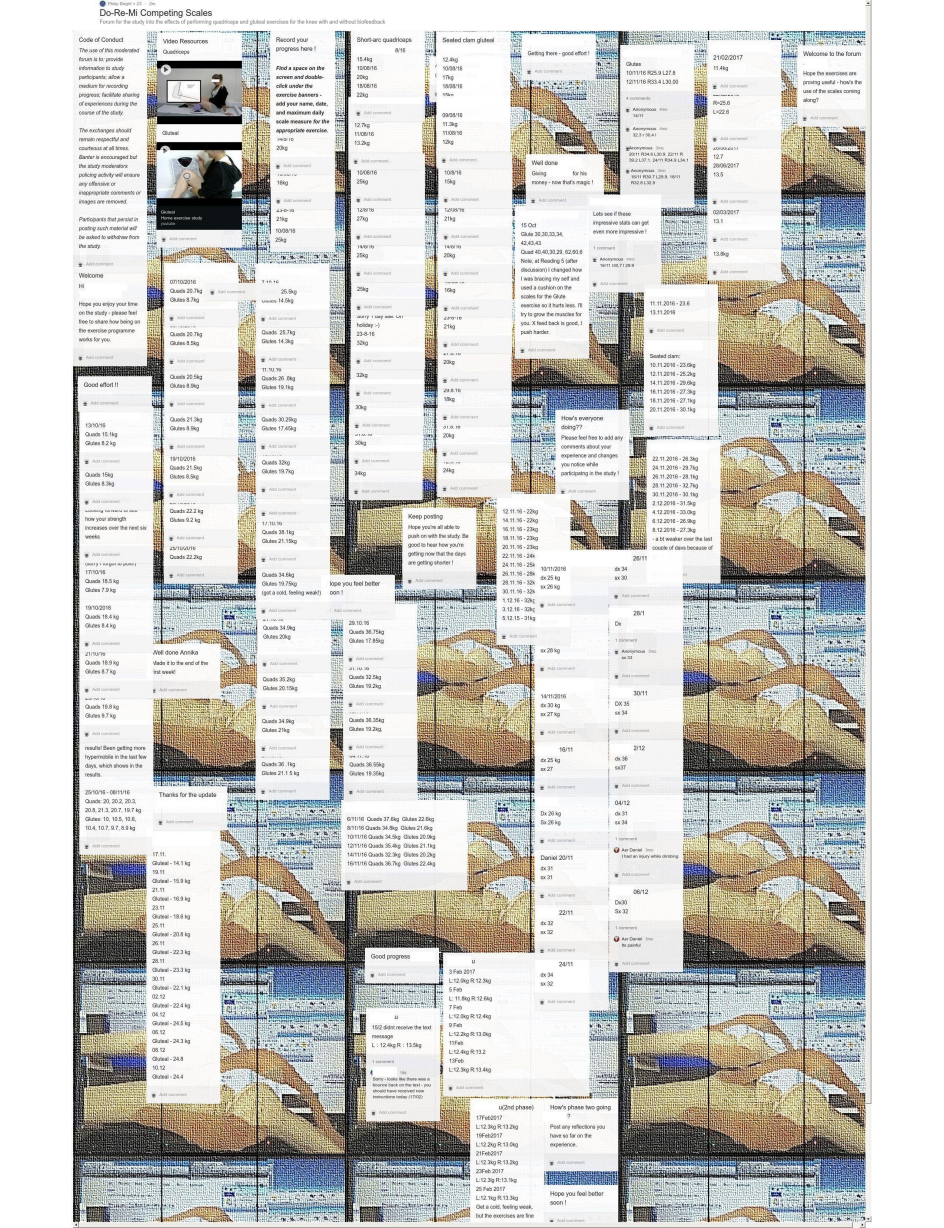
Bespoke Padlet forum with participant and moderator posts.

Twelve individuals (63%) opted to engage with the Padlet forum with entry frequency ranging from 4 to 40. Follow-up on the seven who did not report outcomes elicited four replies; time constraints (n=3) and technophobia (n=1) were cited as reasons for nonresponse. All individuals that initially reported outcomes went on to complete the exercise program regardless of dropout from the forum. The depiction of the finalized notice board entries can be viewed in Figure 3.

Inferential analysis of the influences on reporting by gender and age showed no statistical significance. The odds for male and female responders demonstrated that gender was not a factor in this sample for engaging with the forum activity (OR 0.76, 95% CI 0.06-6.93). There was no significant difference between genders and entry frequency (*P*=.97) or BMI and engagement (*P*=.46). Age and entry frequency also showed no significant correlation (*R*^2^=.054, 95% CI –0.42 to 0.51, *P*=.83).

### Secondary Outcome Measure

Consistent data were reported across all 6 weeks of the study by five of the 12 participants who engaged with the forum (58% attrition rate); three were selected for SPC analysis due to their staggered recruitment dates. The multiple-baseline analysis demonstrated the training effects of participants undertaking the staged exercises and the duration of their engagement with the short arc extension quadriceps exercise.

**Figure 4:**
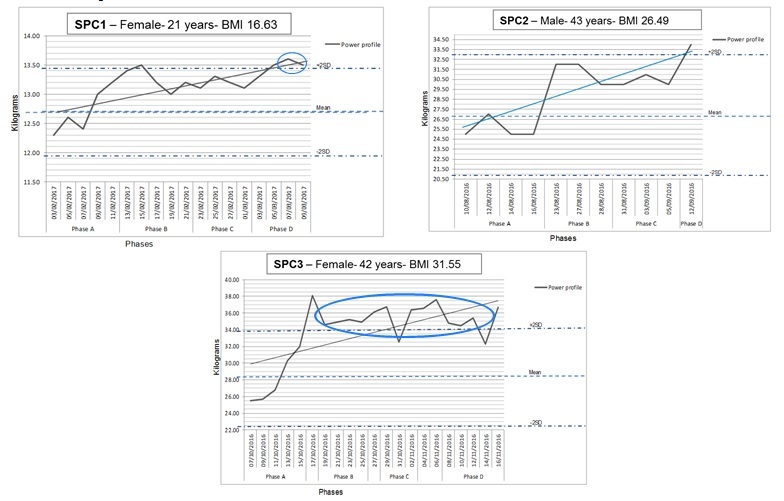
Multiple-baseline analyses of single participant data with statistical thresholds and linear trend lines. SPC: statistical process control.

A progressive conditioning response is demonstrated in Figure 4 with the three line graphs; significant events are depicted in two of three SPC analyses. The first (SPC1) incurs two consecutive data points outside the upper 2 standard deviation threshold at the end of phase D; SPC3 demonstrates a range of significant improvements in reported muscle strength during phase B and D of the study.

### Qualitative Data

Six participants (50%, 6/12) provided limited commentary during their engagement with the online forum; examples are presented in Table 2 that demonstrate themes of pain, mitigation, and response. These participants were representative of the gender (40% female) and age (mean 31, SD 10) of this study’s demographics.

The individuals provided reflection on their experiences and progress in response to the exercises (female, age 22). The mitigating effects of pain were commonly reported in response to perceived decline in performance and reporting (male, age 29). A stoic sense of perseverance was interpreted from the commentary with an adaptation of technical approach when required (female, age 21; male, age 41).

**Table 2:** Illustrative quotes from online forum.

Participant	Theme	Quote
Female, age 22	Mitigation	“Been getting more hypermobile in the last few days, which shows in the results”
Male, age 41	Response	“Feedback is good, I push harder”
	Mitigation/pain	“I changed how I was bracing myself and used a cushion on the scales for the glute exercise so it hurts less”
Female, age 21	Mitigation	“Get a cold, feeling weak but the exercises are fine”
Male, age 42	Mitigation	“A bit weaker over the last couple of days because of flu”
Male, age 29	Pain	“I had an injury while climbing...it’s painful”

## Discussion

The primary aim of this study was to determine the feasibility of patients using an online forum for reporting progress when engaging with a 6-week exercise program for managing knee pain. No statistically significant difference was found in reporting progress based on gender, age, or BMI. It was possible to use individuals’ posted progress data to complete a multiple-baseline case study for a selection of participants in the study. Participants were willing to engage in limited discussion posts during their progression on the program.

Posting to the forum was initially at a moderate level and attrition rates were comparable with other studies exploring engagement with online discussion boards. The 58% reported in this study is in the range of the 12 studies exceeding the 20% attrition rate in the review of Williams et al [24]. Within the scope of behavioral change in eHealth, the range of 41% to 84% attrition is reported in large randomized controlled trials [35]. The consistency of participants’ reports within this study, facilitating individualized progression data, may be indicative of the stable core user remnant that prevails after initial early dropouts [36]. Further exploration of the benefits of self-reporting with the incentive of producing individual activity profiles is warranted, particularly within the scope of affordable technology and activity tracking [37].

Exercise adherence has been identified as a major contributor to exercise efficacy [38]. Participants that made initial engagement with recording their outcomes online committed to the 6-week program irrespective of report attrition. The access to the video instructions through the forum may have influenced this behavior because these media have been seen to improve exercise adherence [39,40]. The growth in interactive video technology may facilitate this further; real-time remote motion capture of patients, tracking, and analyzing movement, with feedback relayed direct from a therapist may be the panacea in this field [41]. There are implications for these types of systems in terms of sensitivity of personal data [42] and developing suitably secure software architecture is an ongoing challenge within the Web 2.0 milieu [43,44]. The integration of body sensor network information into this cloud computing platform and the volume of wearable devices (eg, FitBit, MOOV, Nike+) that can contribute to these biofeedback networks elicits a complex array of data [45]. This potentially lacks meaning or context for patients; the findings of this study demonstrate a simple solution to this complexity.

Age and social media engagement have been reported as conflicting characteristics in studies engaging eHealth with usage mediated by generation. Although engagement activity profiles may differ, those older than 65 years are comparable to those younger than 30 years in terms of the proportions reporting the use of the Internet for health-related information (53% and 56%, respectively) [46]. The age range in this study crossed Generations X and Y, but lacked engagement with senior citizens. Those older than 65 years are motivated to engage with eHealth and increased Internet use as a vital connection with the wider world, offsetting age-related functional changes [47] and physical inactivity [48]. Age was not seen as predictive of engagement in this study, but there is a suggestion that socioeconomic status is an overt influence on Internet use in relation to subjective health [49]. The sample in this study were drawn from undergraduate cohorts but the age span of 19 to 53 years indicates funding sources and social status could not be directly inferred and was not sought at the time of participation.

Gender and BMI may indicate a barrier to information technology use in adolescents and practitioners [50-52], but reported disparities in adoption of Internet-based health correspond more with lower income, educational attainment, ethnic background, and those for whom English is not their native language [53]. Gender and BMI influence on engagement was equivocal in terms of the odds reported in this study; the student sample here may be more consumer-driven, aligned to recent shifts in UK higher education with strong emphasis on student choice and experience and less on gender-based decisions [49]. The shifting engagement in this study’s student participants may be tempered by self-determination and personal preference. Electronic media use has been reported as a risk factor for higher BMI, particularly within the adolescent female population [54]. Conversely, targeted eHealth solutions for weight management in young women suffer from poor uptake and user satisfaction ratings [55]. Activity and diet modification via specialized apps may offer an improved engagement profile around personal weight management in adults [56,57]. Similarly, perceived pressures reported by other health care undergraduates [58] may be applicable to this study and mitigated engagement. Time availability and pressures of course deadlines are also reported as inhibitors to activity-related eHealth [59]. The potential addictive impact of technology and reduced academic performance reported in other studies [60] may have been seen as prohibitive in this study’s sample. Exploration of technology reliance and side effects on prolonged eHealth use is a conflicting relationship that warrants further exploration.

The provision of individualized single-case data fed back to patients contributes to the ideal of personalized, preventive health care planning [61]. The ability for patients to report on their own progress with clinical home-based outcomes has been reported as vital to integrated electronic medical records [62]. The biofeedback information in this study could provide further complementary data to wearable devices [45,63]; this potentially negotiates the pathway between consumer mass adoption and practitioner caution in this developing area [64]. This study demonstrates that patients can have direct access to personal analytics and potentially aid in the management of ongoing conditions. The growing demand to use single-case analyses to inform effect size and meta-evidence [65-67] demands that “big data” from individual patients be used more constructively, particularly the patient-accessible visual analytics afforded within these designs [68].

This study’s sample reported experiences around pain, mitigation, and responsiveness and this was within a recruitment strategy of asymptomatic participants. Subjective and objective pain measures have been widely explored in knee condition sufferers [69,70]. Qualitative data intimates that patients’ outcomes and pain management should be considered on an individual basis [71] with online forums providing the validation, support, and resources as required [28]. The sample in this study described mitigating effects of pain in relation to the exercise task orientation. This contrasts with young symptomatic individuals that report the burden of musculoskeletal pain on quality of life and future prospects; the need for digital technologies to provide accessible, evidence-based resources is seen as vital in connecting these people with support from peers and health professionals [72]. The individuals in this study were potentially engaging from a sense of duty and felt compelled to offer mitigation when compliance wavered. There is suggestion that compelling pain management programs may only arise with a population that perceives the need for individualized care, particularly if that population feels disenfranchised [73].

Limitations of this study include selection bias with a convenience sample of undergraduate students. Only those prepared to commit to the program were included indicating that participants had an underlying motivation toward exercise. All participants were asymptomatic implicating the diversity in compliance; attrition could be further mitigated with a motivated symptomatic patient population. The extension to engage with people older than 65 years in future studies would allow the development of this type of online health community in condition-specific scenarios. Socioeconomic status was not captured by this study and this is seen as a key influence on access and engagement in the field of eHealth; such barriers to engagement have to be explored further. This study was able to demonstrate that a low-cost solution to developing an online health community is feasible and that individualized, patient-centric data can be produced from reporting biofeedback data on an online forum. Future research should look to investigate discordance between attitudes to technology-assisted health care, the importance of individualized visual data to patients, and the role of forums in monitoring patient engagement and progress in symptomatic populations.

Patients can engage with an online forum for reporting progress when complying with exercise programs for managing knee pain. No significant influence was found on reporting progress in an online forum based on gender, age, or BMI. It was possible to use individual posted progress data to complete a multiple-baseline case study for a selection of participants in the study. Participants were willing to engage in limited discussion posts during their progression on the program. The parochial nature of the sample is a limitation; future work in the area should look to address discordance between attitudes to technology-assisted health care, the importance of individualized visual data to patients, and the role of forums in monitoring patient engagement and progress in symptomatic knee pain populations. Socioeconomic background and other barriers to accessing these community forums need to be considered in this exploration.

## Appendix

Multimedia Appendix 1 Powerpoint presentation of study.

## Abbreviations

BMI: body mass index
SPC: statistical process control
