# Supplementary material for: Patients Using an Online Forum for Reporting Progress When Engaging With a Six-Week Exercise Program for Knee Conditioning: Feasibility Study
Source: JMIR Rehabil Assist Technol. 2018 Apr 26;5(1):e9. doi: 10.2196/rehab.8567 (PMC5945989; doi:10.2196/rehab.8567)
Supplement: Multimedia Appendix 1 [file rehab_v5i1e9_app1.pptx]

## Slide 1
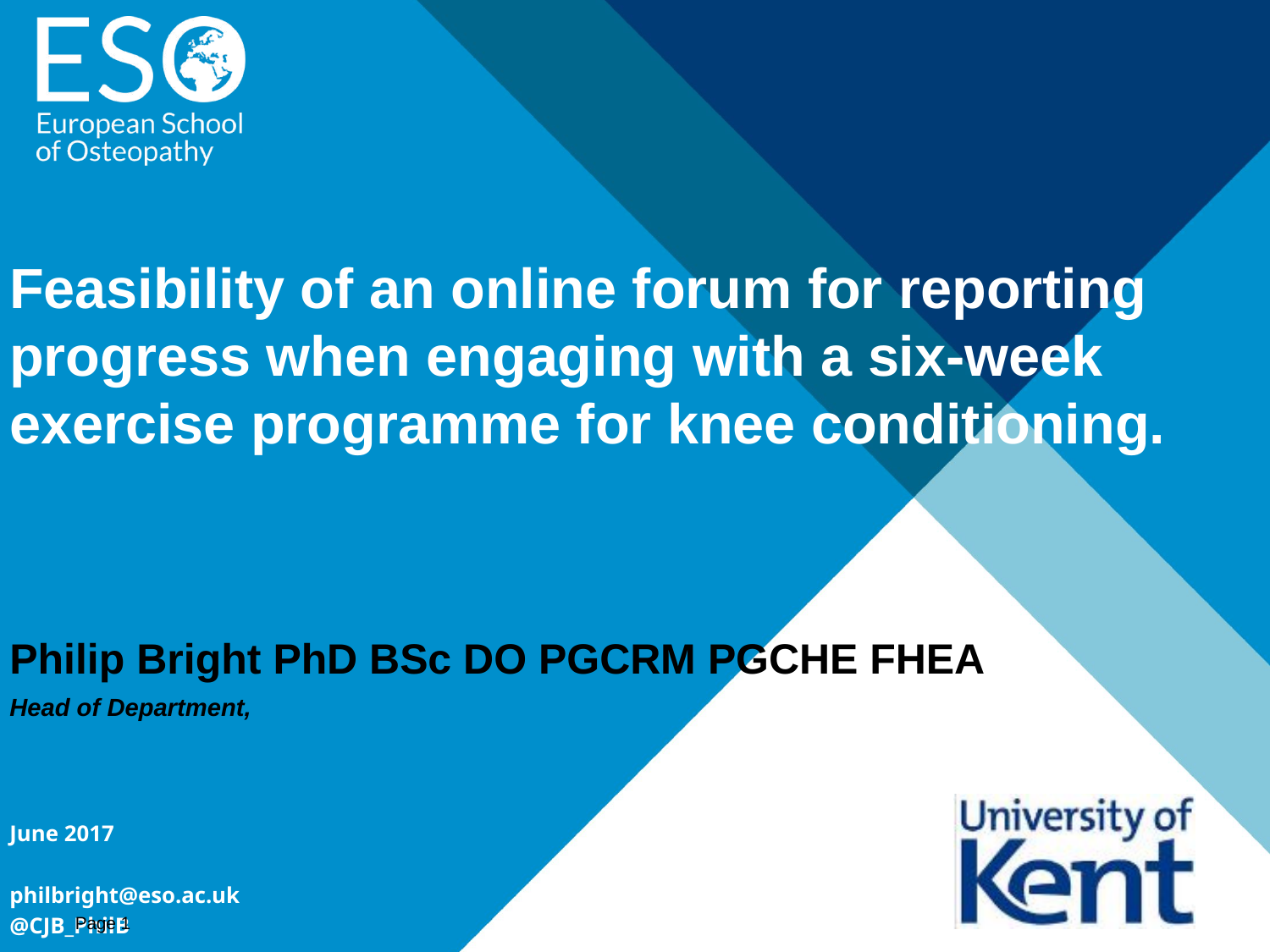

Feasibility of an online forum for reporting progress when engaging with a six-week exercise programme for knee conditioning.
Philip Bright PhD BSc DO PGCRM PGCHE FHEA
Head of Department,
June 2017
philbright@eso.ac.uk
@CJB_PhilB
Page 1

## Slide 2
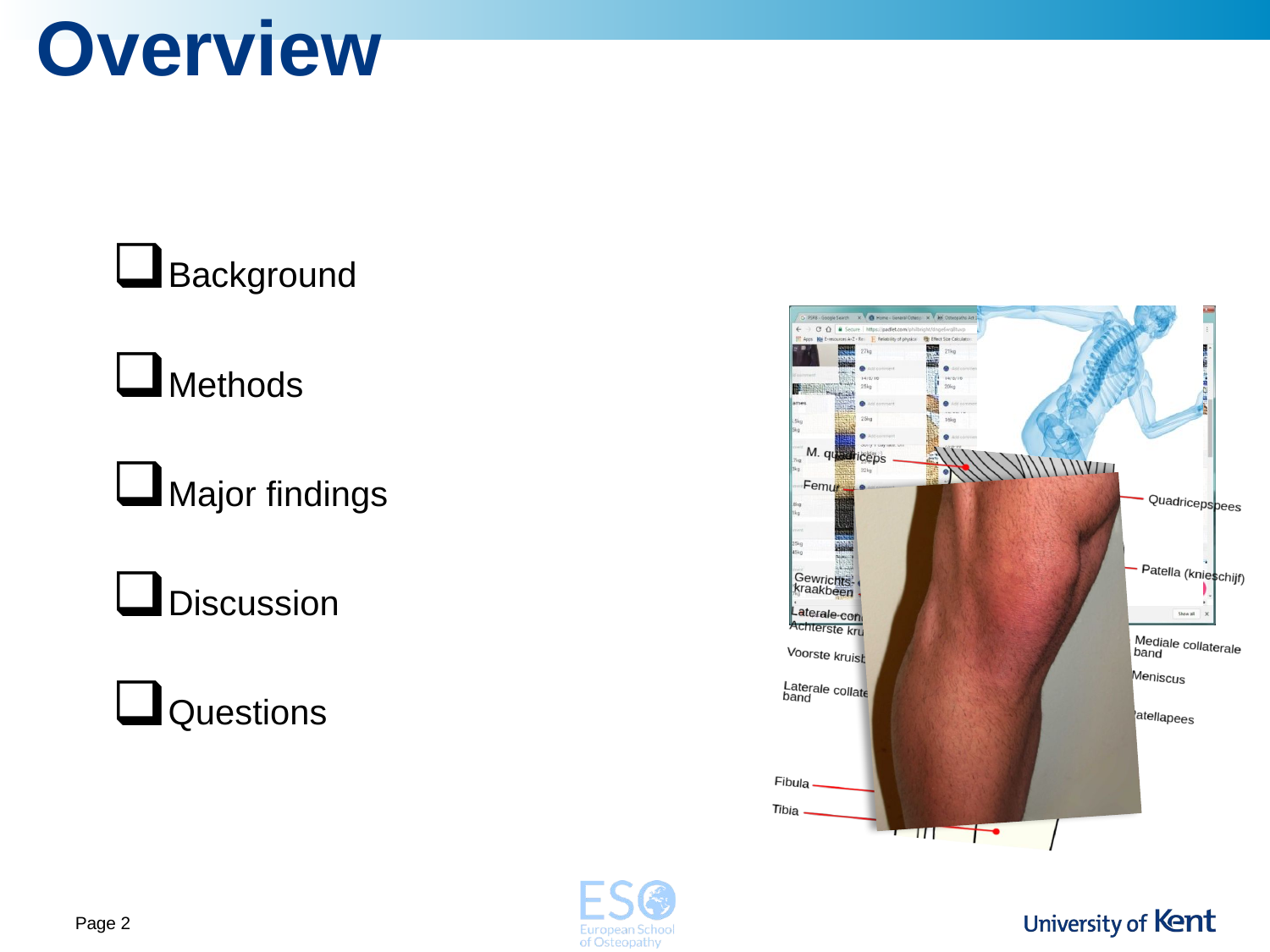

# Overview
Background
Methods
Major findings
Discussion
Questions
Page 2

## Slide 3
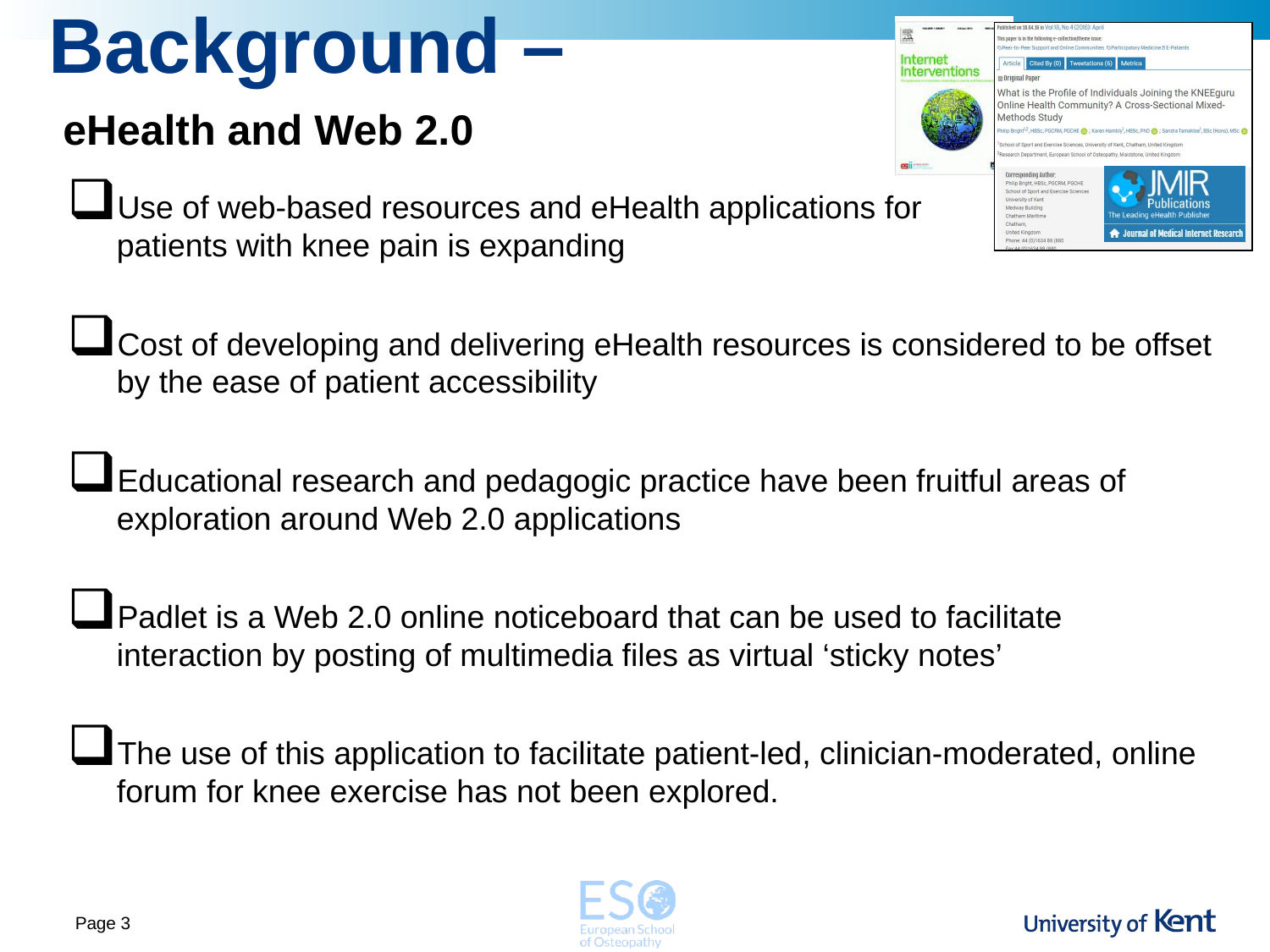

Background –
eHealth and Web 2.0
Use of web-based resources and eHealth applications for 		 patients with knee pain is expanding
Cost of developing and delivering eHealth resources is considered to be offset by the ease of patient accessibility
Educational research and pedagogic practice have been fruitful areas of exploration around Web 2.0 applications
Padlet is a Web 2.0 online noticeboard that can be used to facilitate interaction by posting of multimedia files as virtual ‘sticky notes’
The use of this application to facilitate patient-led, clinician-moderated, online forum for knee exercise has not been explored.
Page 3

## Slide 4
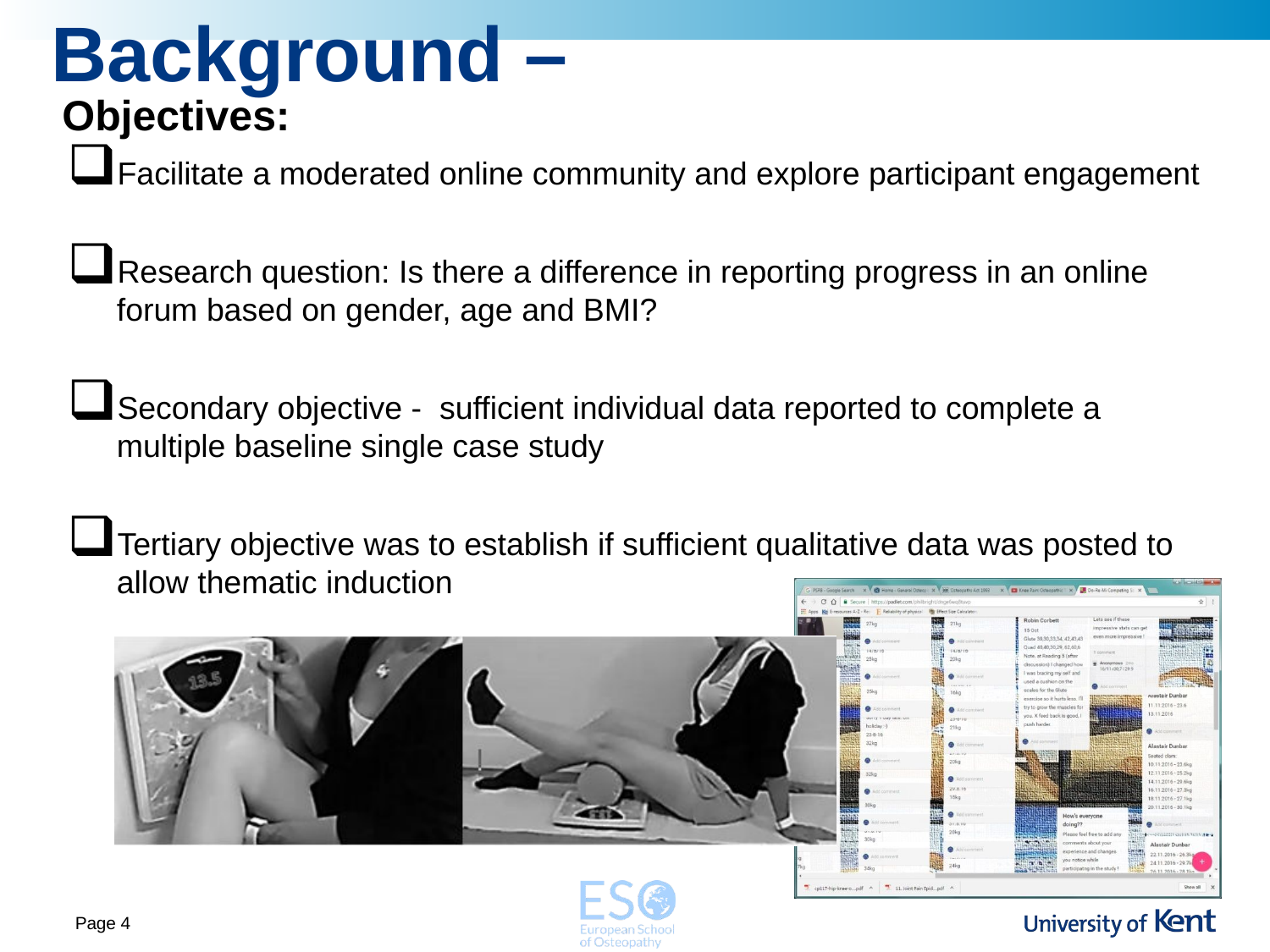

Background –
Objectives:
Facilitate a moderated online community and explore participant engagement
Research question: Is there a difference in reporting progress in an online forum based on gender, age and BMI?
Secondary objective - sufficient individual data reported to complete a multiple baseline single case study
Tertiary objective was to establish if sufficient qualitative data was posted to allow thematic induction
Page 4

## Slide 5
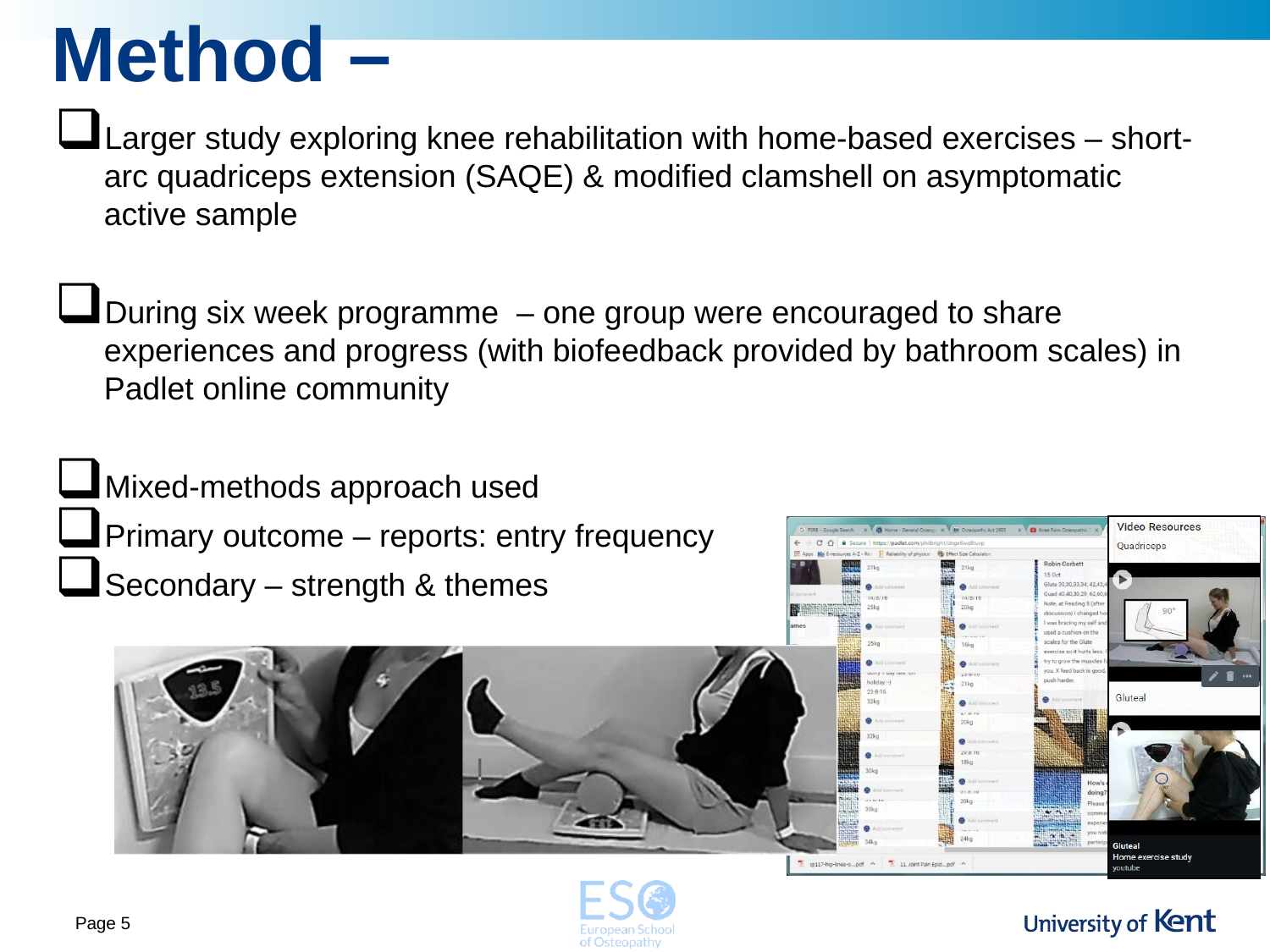

Method –
Larger study exploring knee rehabilitation with home-based exercises – short-arc quadriceps extension (SAQE) & modified clamshell on asymptomatic active sample
During six week programme – one group were encouraged to share experiences and progress (with biofeedback provided by bathroom scales) in Padlet online community
Mixed-methods approach used
Primary outcome – reports: entry frequency
Secondary – strength & themes
Page 5

## Slide 6
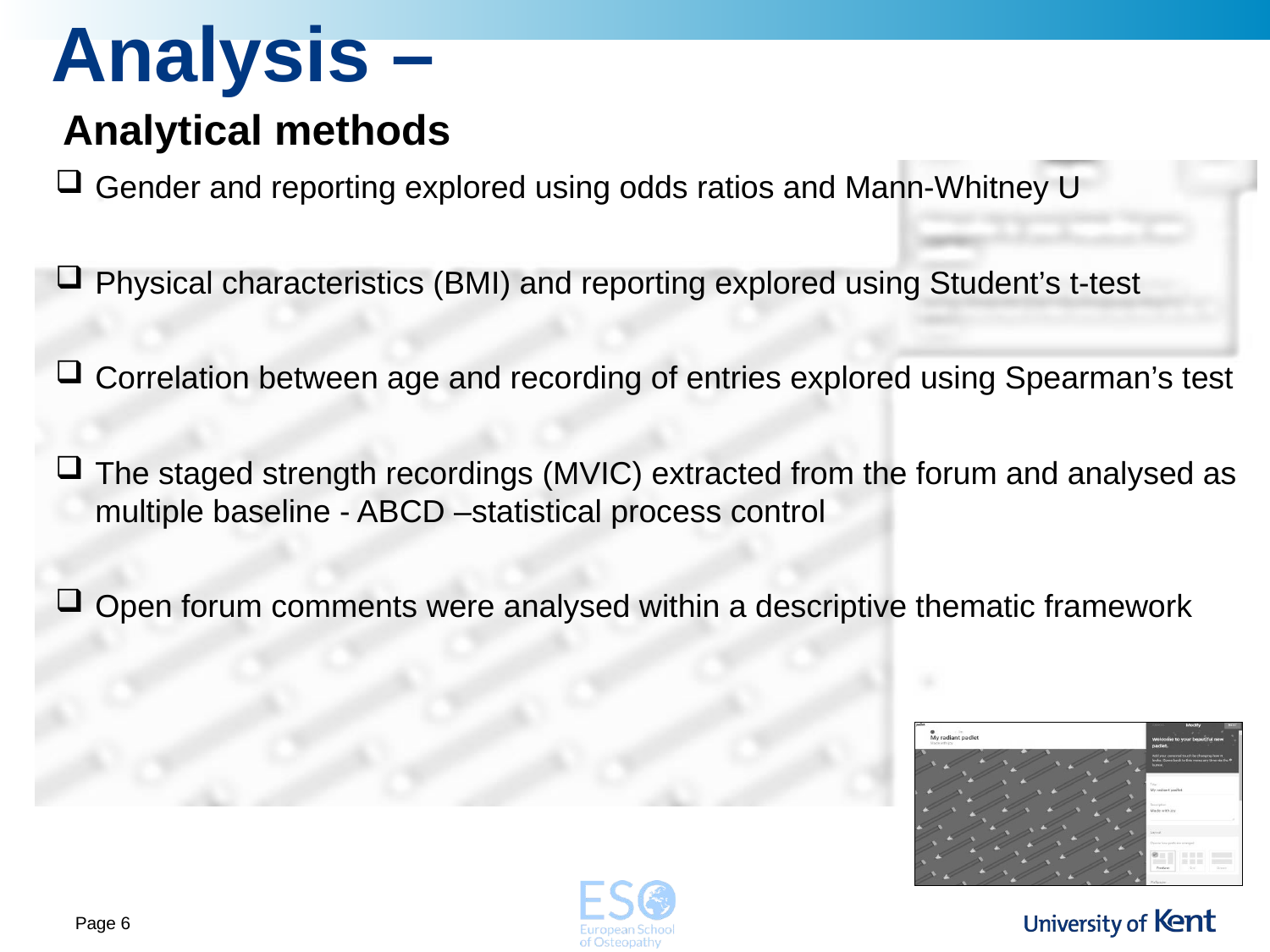

Analysis –
Analytical methods
Gender and reporting explored using odds ratios and Mann-Whitney U
Physical characteristics (BMI) and reporting explored using Student’s t-test
Correlation between age and recording of entries explored using Spearman’s test
The staged strength recordings (MVIC) extracted from the forum and analysed as multiple baseline - ABCD –statistical process control
Open forum comments were analysed within a descriptive thematic framework
Page 6

## Slide 7
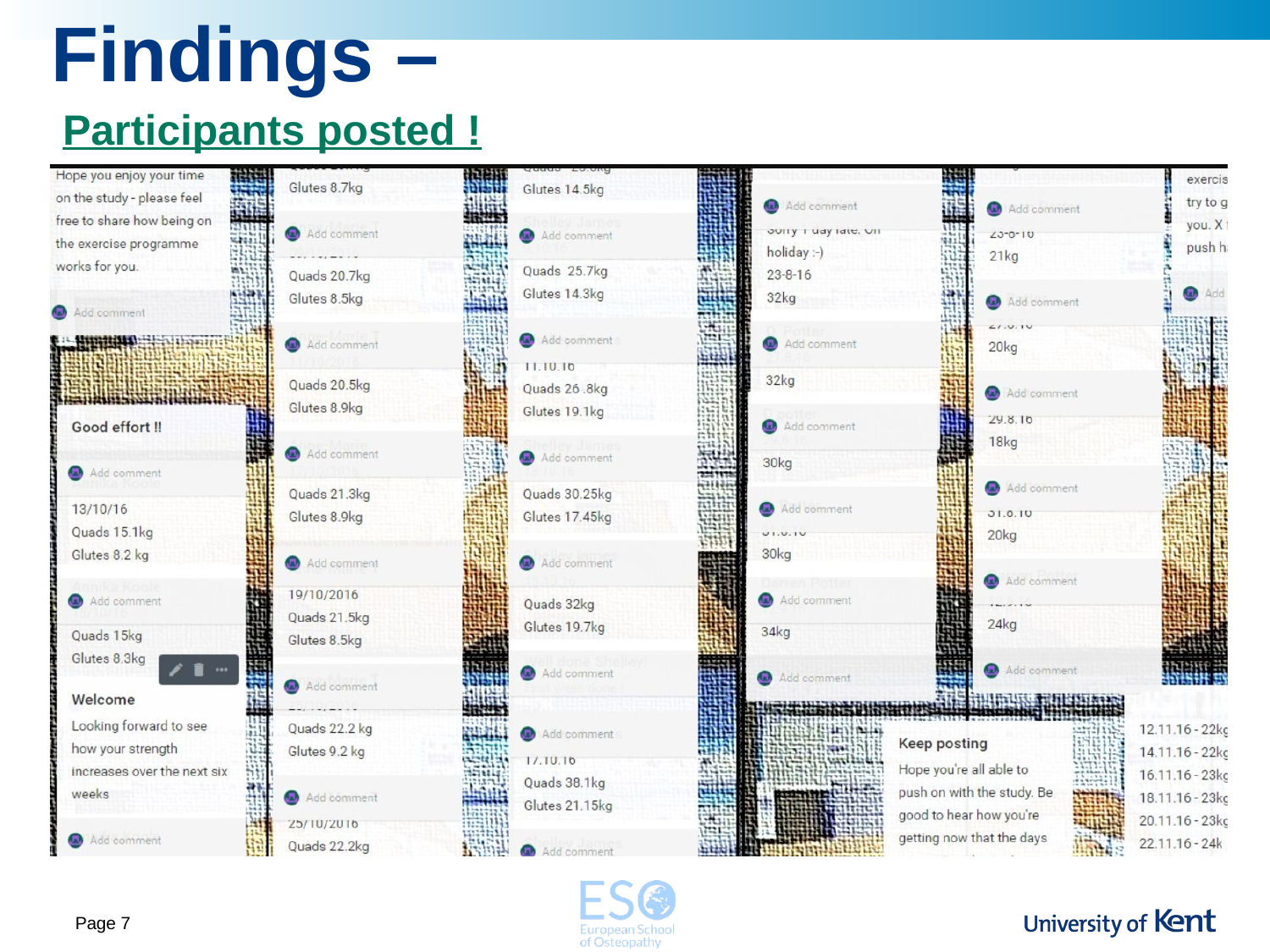

Findings –
Participants posted !
Page 7

## Slide 8
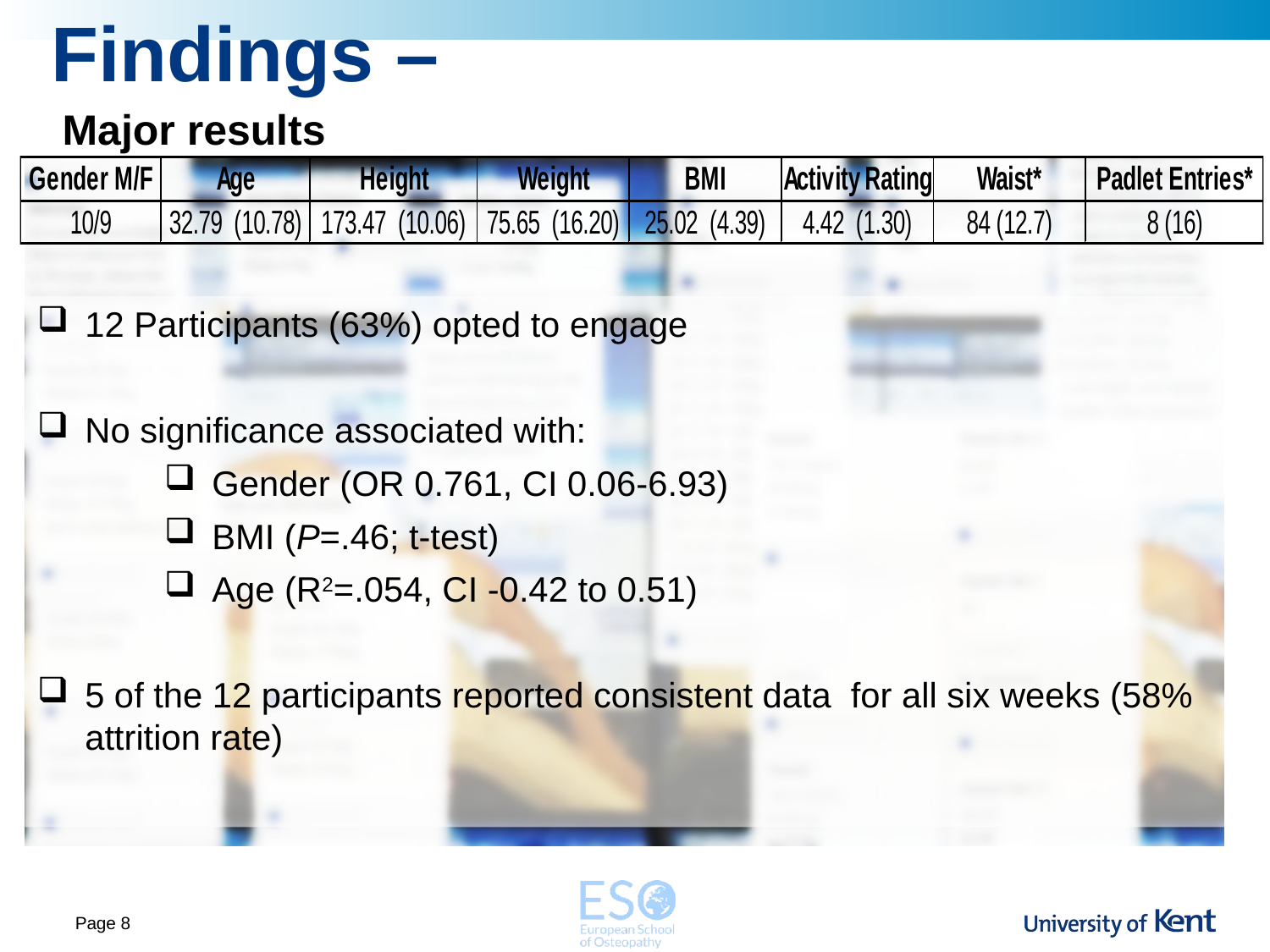

Findings –
Major results
12 Participants (63%) opted to engage
No significance associated with:
Gender (OR 0.761, CI 0.06-6.93)
BMI (P=.46; t-test)
Age (R2=.054, CI -0.42 to 0.51)
5 of the 12 participants reported consistent data for all six weeks (58% attrition rate)
Page 8

## Slide 9
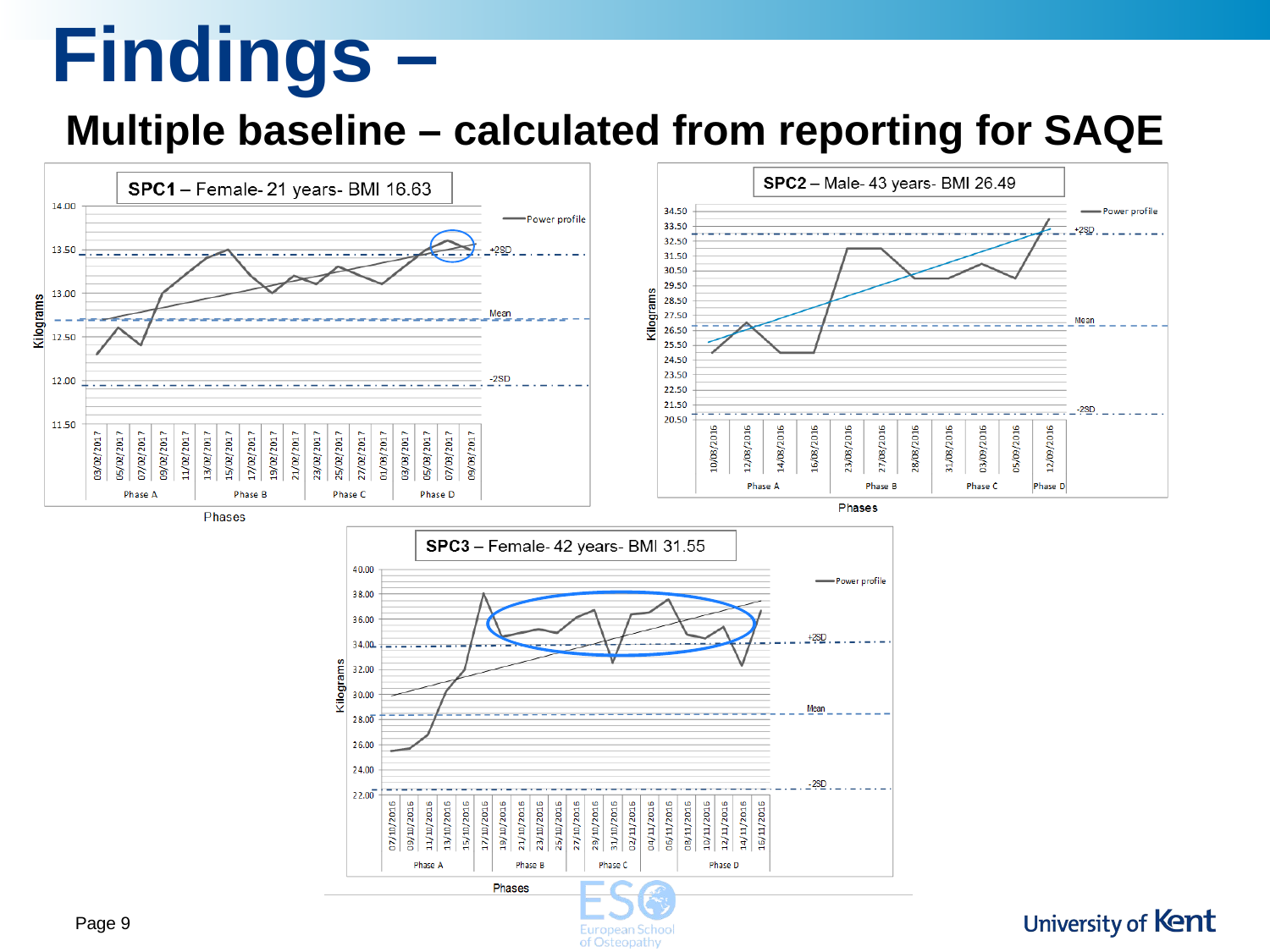

Findings –
Multiple baseline – calculated from reporting for SAQE
Page 9

## Slide 10
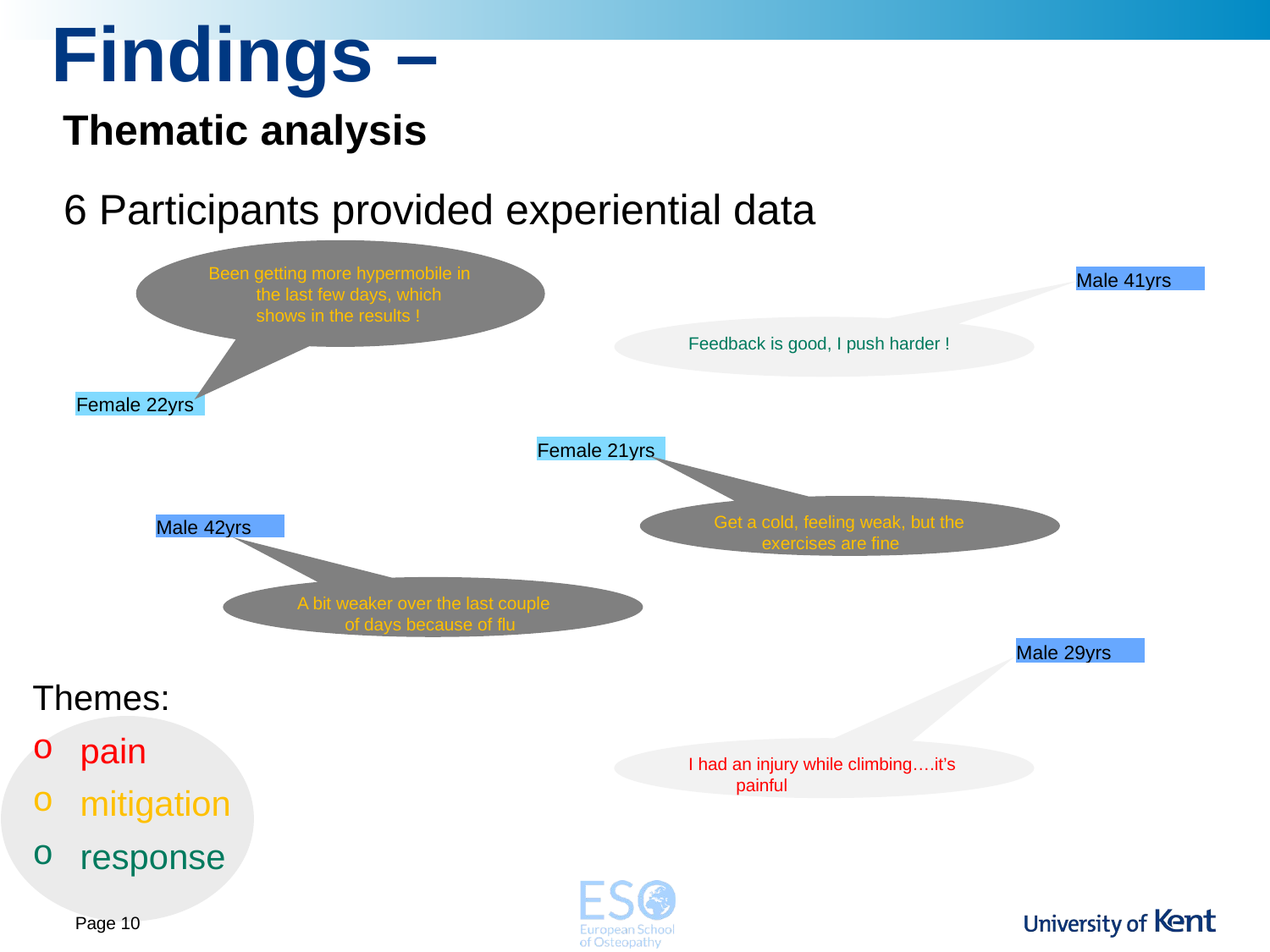

Findings –
Thematic analysis
6 Participants provided experiential data
Been getting more hypermobile in the last few days, which shows in the results !
| Male 41yrs |
| --- |
Feedback is good, I push harder !
| Female 22yrs |
| --- |
| Female 21yrs |
| --- |
Get a cold, feeling weak, but the exercises are fine
| Male 42yrs |
| --- |
A bit weaker over the last couple of days because of flu
Themes:
pain
mitigation
response
| Male 29yrs |
| --- |
I had an injury while climbing….it’s painful
Page 10

## Slide 11
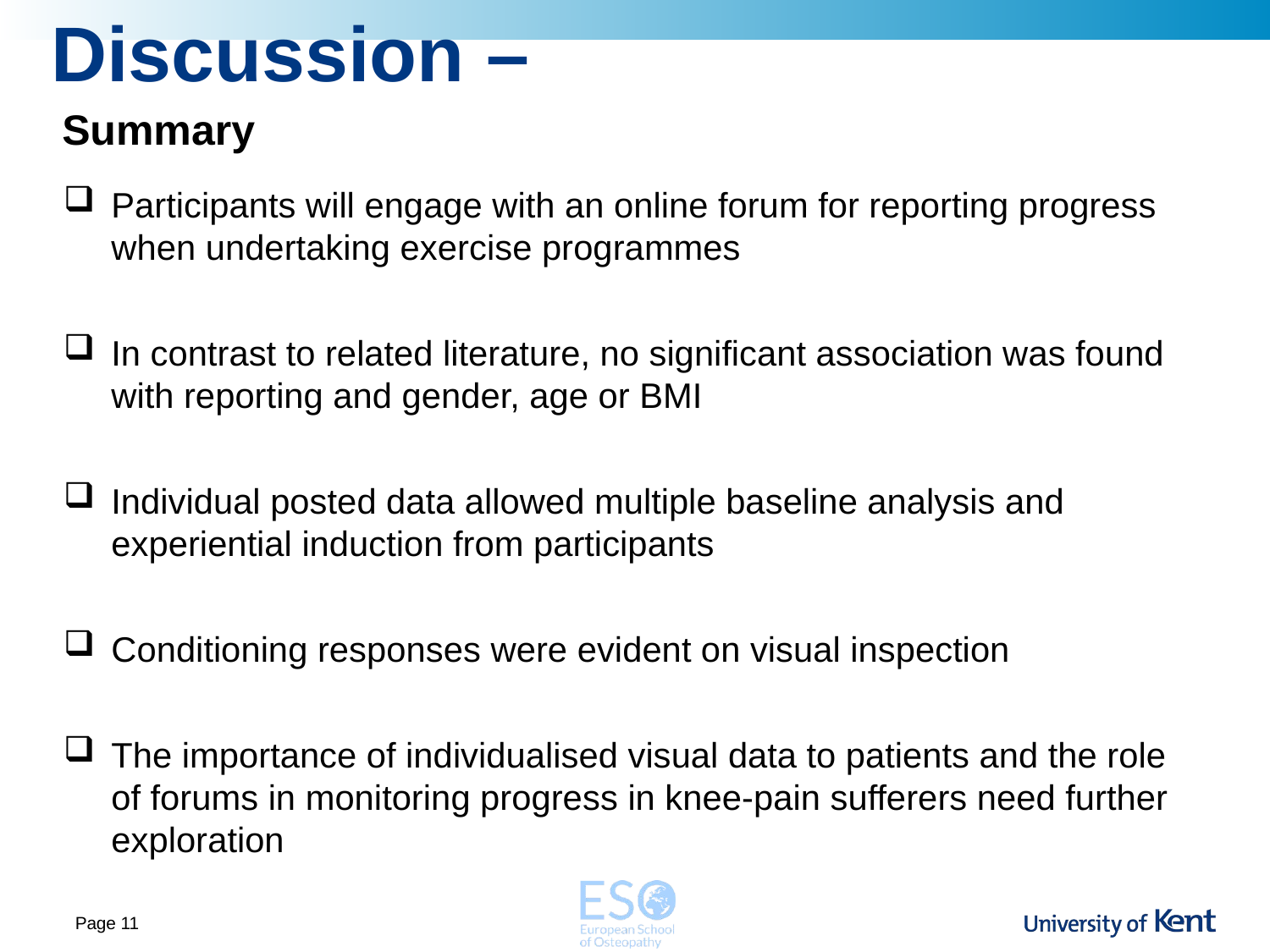

Discussion –
Summary
Participants will engage with an online forum for reporting progress when undertaking exercise programmes
In contrast to related literature, no significant association was found with reporting and gender, age or BMI
Individual posted data allowed multiple baseline analysis and experiential induction from participants
Conditioning responses were evident on visual inspection
The importance of individualised visual data to patients and the role of forums in monitoring progress in knee-pain sufferers need further exploration
Page 11

## Slide 12
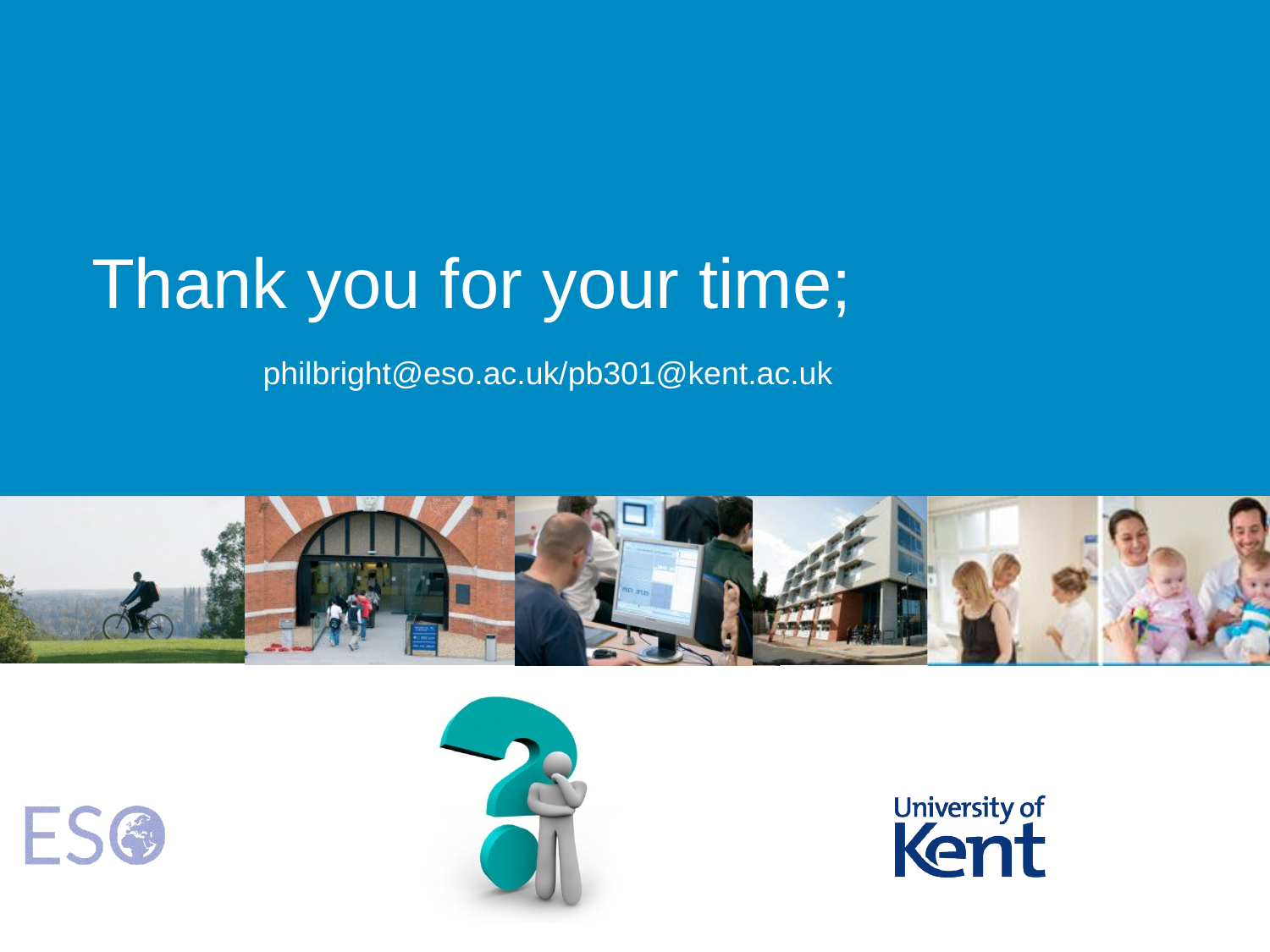

# Thank you for your time; philbright@eso.ac.uk/pb301@kent.ac.uk
